# Preventing PTSD with oxytocin: effects of oxytocin administration on fear neurocircuitry and PTSD symptom development in recently trauma-exposed individuals

**DOI:** 10.1080/20008198.2017.1302652

**Published:** 2017-04-11

**Authors:** Jessie L. Frijling

**Affiliations:** ^a^ Department of Psychiatry, Academic Medical Center, University of Amsterdam, Amsterdam, the Netherlands

**Keywords:** PTSD, early intervention, prevention, oxytocin, amygdala, fMRI

## Abstract

**Background**: Posttraumatic stress disorder (PTSD) is a debilitating psychiatric disorder which develops in approximately 10% of trauma-exposed individuals. Currently, there are few early preventive interventions available for PTSD. Intranasal oxytocin administration early posttrauma may prevent PTSD symptom development, as oxytocin administration was previously found to beneficially impact neurobiological (e.g. amygdala reactivity) and socio-emotional PTSD vulnerability factors.

**Objective:** The overall aim of this dissertation was to investigate the potential of intranasal oxytocin administration as early preventive intervention for PTSD.

**Methods**: We performed a functional magnetic resonance imaging (fMRI) study to assess the acute effects of a single administration of oxytocin on the functional fear neurocircuitry – consisting of the amygdala and (pre)frontal brain regions – in recently trauma-exposed emergency department patients (range *n *= 37–41). In addition, we performed a multicentre randomized double-blind placebo-controlled clinical trial (RCT) to assess the efficacy of repeated intranasal oxytocin administration early after trauma for preventing PTSD symptom development up to six months posttrauma (*n *= 107).

**Results**: In our fMRI experiments we observed acutely increased amygdala reactivity to fearful faces and attenuated amygdala-ventromedial and ventrolateral prefrontal cortex functional connectivity after a single oxytocin administration in recently trauma-exposed individuals. However, in our RCT we found that repeated intranasal oxytocin administration early posttrauma reduced subsequent PTSD symptom development in recently trauma-exposed emergency department patients with high acute PTSD symptoms.

**Conclusions**: These findings indicate that repeated intranasal oxytocin is a promising early preventive intervention for PTSD for individuals at increased risk for PTSD due to high acute symptom severity. Administration frequency dependent effects of oxytocin or the effects of oxytocin administration on salience processing may serve as explanatory frameworks for the contrasting oxytocin effects on anxiety-related measures in our clinical and neuroimaging studies.

## 1. Introduction

Exposure to psychological trauma is a common phenomenon, as approximately 70% of the worldwide general population experiences at least one traumatic event during their lifetime (Benjet et al., 2015). Approximately 10% of trauma-exposed individuals develops **posttraumatic stress disorder (PTSD)** (de Vries & Olff, 2009; Kessler, Petukhova, Sampson, Zaslavsky, & Wittchen, 2012). PTSD is not only a burden for the affected individual and his/her immediate surroundings, but may also be considered a public health issue (Magruder, Kassam-Adams, Thoresen, & Olff, 2016). PTSD is associated with the highest health care costs compared to other anxiety disorders (Marciniak et al., 2005), which even further increase in case of comorbid major depression disorder or substance use disorder, which are both highly prevalent in PTSD patients (Kessler et al., 2012; Marciniak et al., 2005; McCauley, Kileen, Gros, Brady, & Back, 2012).

Evidence-based treatments for PTSD include trauma-focused psychotherapy (e.g. cognitive behaviour therapy (CBT), eye movement desensitization reprocessing (EMDR)) and treatment with selective serotonin reuptake inhibitors (SSRIs) (Forbes et al., 2010). However, in a study in the general population, almost 93% of PTSD patients did not seek treatment within the first year of symptoms (Wang et al., 2005). Furthermore, these therapies are not sufficiently effective in about one-third of patients (Bradley, Greene, Russ, Dutra, & Westen, 2005; Pull & Pull, 2014). Therefore, substantial effort should be made to minimize the individual and societal burden associated with PTSD (Olff, Van Zuiden, & Bakker, 2015).

**Secondary prevention of PTSD** may reduce the burden associated with PTSD, as effective prevention results in fewer trauma-exposed individuals developing (chronic) PTSD. As trauma exposure constitutes an identifiable event of potential PTSD onset, the first hours to weeks posttrauma serve as a suitable time-period for the provision of early preventive interventions for PTSD, aimed to reduce subsequent PTSD symptom development. Previously, administration of benzodiazepines (Gelpin, Bonne, Peri, Brandes, & Shalev, 1996; Mellman, Bustamante, David, & Fins, 2002), SSRIs (Galatzer-Levy et al., 2013) and the beta-adrenergic receptor blocking agent propranolol (Hoge et al., 2012; Stein, Kerridge, Dimsdale, & Hoyt, 2007) early posttrauma did not reduce subsequent PTSD symptom development in recently trauma-exposed individuals. Similarly, psychological debriefing within the first weeks posttrauma was ineffective in preventing subsequent PTSD; there are even indications that debriefing increased PTSD symptoms in some individuals (Rose, Bisson, Churchill, & Wessely, 2002; Sijbrandij, Olff, Reitsma, Carlier, & Gersons, 2006). Current early interventions that hold significant promise for effective PTSD prevention, i.e. hydrocortisone administration (Delahanty et al., 2013; Zohar et al., 2011), prolonged exposure therapy (Rothbaum et al., 2012) and brief CBT (Roberts, Kitchiner, Kenardy, & Bisson, 2009), are not yet at the stage of widespread implementation, as clinical evidence supporting their efficacy is still sparse (Sijbrandij, Kleiboer, Bisson, Barbui, & Cuijpers, 2015). Therefore, it remains highly relevant to further study novel preventive interventions for PTSD.

One potential strategy for novel preventive interventions for PTSD is to pharmacologically target neurobiological and socio-emotional risk factors for PTSD early posttrauma that are likely to be etiologically involved in PTSD development. In recent years, it was observed that autonomic and glucocorticoid reactivity to stress, assessed prior to or early posttrauma, predicted PTSD symptom development (Coronas et al., 2011; Pole et al., 2009; van Zuiden, Kavelaars, Geuze, Olff, & Heijnen, 2013). Also, pre- and early posttrauma hyperreactivity of the amygdala – a brain region with a pivotal role in detection, expression, regulation and learning of fear – was found to predict subsequent PTSD symptoms (Admon et al., 2009; McLaughlin et al., 2014). Furthermore, low perceived social support early posttrauma has been associated with increased PTSD risk (Brewin, Andrews, & Valentine, 2000). Intranasal administration of the neuropeptide oxytocin (see Section 1.1 for additional background information) is a promising pharmacological agent for PTSD prevention (Olff, 2012). Accumulating evidence from studies in animals and in human populations with and without psychiatric disorders shows that oxytocin administration may modulate autonomic stress reactivity (Kubzansky, Mendes, Appleton, Block, & Adler, 2012), attenuate anxiety and amygdala reactivity (Kirsch et al., 2005; Koch et al., 2016b), as well as beneficially impact socio-emotional processes and pro-social behaviour (Preckel, Scheele, Kendrick, Maier, & Hurlemann, 2014; van IJzendoorn & Bakermans-Kranenburg, 2012). Given these beneficial effects of oxytocin administration on factors associated with increased PTSD risk, it was hypothesized that administration of intranasal oxytocin early posttrauma may prevent subsequent PTSD symptom development (Olff, 2012).

Oxytocin

Oxytocin is a mammalian neuropeptide and is synthesized in the periventricular and supraoptic nuclei of the hypothalamus. It is released into the circulation from the posterior pituitary. Neuronal projections from the hypothalamus send oxytocin to other brain regions. In humans, brain regions that express the oxytocin receptor – and thereby are likely affected by oxytocin – include the central and basolateral amygdala, brainstem, olfactory nucleus, and anterior cingulate cortex (Boccia, Petrusz, Suzuki, Marson, & Pedersen, 2013). In rest, peripheral oxytocin levels are comparable between men and women (de Jong et al., 2015; Feldman, Gordon, Schneiderman, Weisman, & Zagoory-Sharon, 2010; Graugaard-Jensen, Hvistendahl, Frøkiaer, Bie, & Djurhuus, 2014).

Oxytocin is physiologically involved in parturition and lactation by acting as a smooth muscle contractor on the uterus and lactation glands. In the 1990s, pioneering work in prairie voles – socially monogamous rodents that are similar to humans in their tendency and ability to form strong social pair bonds – demonstrated that oxytocin is a crucial neurobiological mediator of social pair bond formation and maternal behaviour. Subsequently, studies in humans demonstrated oxytocin’s involvement in trust, attachment, empathy, paternal behaviour and romantic relationships (Feldman, 2012). As a result, popular media – and also scientists (Carter, 1998; Neumann, 2007; Olff, Van Zuiden, Koch, et al., 2015) – often reference to oxytocin as the ‘cuddle’, ‘love’, or ‘miracle’ hormone. As oxytocin also appeared to have anti-stress (Cardoso, Kingdon, & Ellenbogen, 2014; Heinrichs, Baumgartner, Kirschbaum, & Ehlert, 2003) and anxiolytic properties (Huber, Veinante, & Stoop, 2005; Kirsch et al., 2005; Knobloch et al., 2012; Viviani et al., 2011) – and most psychiatric disorders are associated with both social dysfunction and aberrant stress reactivity – the attention of researchers within psychiatry was definitely drawn. A novel research field grew investigating potential clinical applications of intranasal oxytocin administration in psychiatric populations. This has not only yielded a large body of studies on intranasal oxytocin effects on a wide variety of socio-emotional and neurobiological outcome measures, but has also resulted in more critical, nuanced views of the clinical potential of intranasal oxytocin administration (Carson, Yuan, & Labuschagne, 2016; Leng & Ludwig, 2016), compared to the early overly positive views – or hopes – regarding the effects of intranasal oxytocin administration for clinical purposes.

## 2. Aims

The overall aim of this dissertation was to investigate the potential of intranasal oxytocin administration as early preventive intervention for PTSD, by assessing the effects of intranasal oxytocin early after trauma on acute functioning of the fear neurocircuitry and on PTSD symptom development in recently trauma-exposed individuals. To this end, we performed a functional magnetic resonance imaging (fMRI) study to assess the acute effects of a single administration of oxytocin on the functional fear neurocircuitry – consisting of the amygdala and (pre)frontal brain regions – in recently trauma-exposed emergency department patients. In addition, we performed a randomized controlled clinical trial to assess the efficacy of repeated intranasal oxytocin administration early after trauma for preventing PTSD symptom development.

## 3. Summary of findings

### 3.1. Part I – background

In chapter 2 (Frijling et al., 2014b) we discussed the rationale behind our hypothesis that intranasal oxytocin administration may prevent the development of PTSD symptoms. Increased risk for PTSD development is associated with low levels of perceived social support, high heart rate, low heart rate variability, low cortisol levels, and high amygdala reactivity to negative stimuli as assessed early posttrauma. Modification of these vulnerability factors early posttrauma may reduce PTSD risk. A large body of evidence suggests that autonomic and glucocorticoid stress reactivity, neural fear responsiveness and social behaviour are all beneficially affected by endogenous oxytocin and (a single) oxytocin administration. Therefore, risk for PTSD may be attenuated by stimulating the oxytocin system in recently trauma-exposed individuals, for example by administering intranasal oxytocin.

In chapter 3 (Olff et al., 2013) we described that oxytocin administration may improve mental health, as oxytocin administration appears to beneficially affect stress regulation and social behaviour. However, we showed based on previously published findings and novel experimental data that effects of oxytocin administration depend on context and interindividual differences. We argued that such differential effects of oxytocin administration may be related to oxytocin administration enhancing salience processing. Increased salience processing refers to increased processing of contextual cues that normally attract attention (i.e. emotional facial expressions, danger signals). Therefore, although evidence suggests that oxytocin administration may promote mental health given its important role in regulating social behaviour and stress, context and interindividual differences that influence the effects of oxytocin administration should be carefully considered in clinical studies on intranasal oxytocin.

### 3.2. Part II – functional neuroimaging studies

We assessed the acute effects of a single administration of intranasal oxytocin on the fear neurocircuitry in a randomized double-blind placebo-controlled functional magnetic resonance imaging (fMRI) study in trauma-exposed emergency department patients within 11 days posttrauma. In chapter 4 (Frijling et al., 2016a) we assessed the effects of oxytocin administration on amygdala reactivity to emotional faces, using a face-matching task with fearful, happy and neutral faces (*n* = 41). We observed that the effects of oxytocin administration on amygdala reactivity to emotional faces depended on stimulus valence and on sex. In all participants, oxytocin administration increased amygdala reactivity to fearful faces. Additionally, in women only, oxytocin administration increased amygdala reactivity to neutral faces. Our exploratory analysis showed that acute PTSD symptom severity was not associated with differential intranasal oxytocin administration effects on amygdala reactivity. Given these results we argued that a single intranasal administration of oxytocin may increase neural fear processing, possibly as a result of oxytocin-induced enhanced salience processing.

In chapter 5 (Frijling et al., 2016b) we investigated oxytocin administration effects on amygdala-centred emotion and salience network functional connectivity after a trauma reminder, using neutral and trauma script-driven imagery (*n* = 37). For each participant two resting-state fMRI scans were acquired: one after listening to a personal neutral script, and the second after listening to a personal trauma script which was based on the recent traumatic event. We observed that oxytocin-treated participants had diminished amygdala-left ventrolateral prefrontal cortex (vlPFC) functional connectivity in response to the trauma script compared to the neutral script, whereas an increase in amygdala-left vlPFC functional connectivity was observed in placebo-treated participants. In addition, irrespective of script condition, oxytocin administration enhanced amygdala-left (posterior) insula functional connectivity and decreased amygdala-ventromedial prefrontal cortex (vmPFC) functional connectivity. These neural oxytocin administration effects were accompanied by lower levels of sleepiness and higher flashback intensity after the trauma script in oxytocin-treated participants. Taken together, these observations indicate that that a single intranasal oxytocin administration may acutely impede emotion regulation in recently trauma-exposed individuals.

### 3.3. Part III – randomized controlled clinical trial

Chapter 6 (Frijling et al., 2014a) describes the study protocol of our randomized double-blind placebo-controlled clinical trial, in which we investigated the effects of repeated intranasal oxytocin administration on PTSD symptom development in distressed recently trauma-exposed emergency department patients. The primary aim was to investigate the effects of an eight-day intranasal oxytocin treatment regimen initiated within 12 days posttrauma on PTSD symptoms at 1.5 month posttrauma. One of our secondary aims was to assess the effects of repeated oxytocin administration on PTSD, depression and anxiety symptoms at 1.5, three and six months posttrauma. We additionally investigated whether baseline characteristics moderated the effects of repeated oxytocin administration on symptom severity scores, given previous observations that oxytocin administration effects depend on interindividual differences.

In chapter 7 (van Zuiden et al., 2016) the results of our clinical trial are presented. We demonstrated that there was no overall effect of repeated oxytocin administration on PTSD, depression and anxiety symptoms up to six months posttrauma (*n* = 107). However, the effects of oxytocin administration were moderated by acute PTSD symptom severity assessed prior to the intervention. Oxytocin administration reduced PTSD symptoms up to six months posttrauma in trauma-exposed individuals with high acute PTSD symptoms only. These findings suggest that an eight-day intranasal oxytocin treatment regimen is a promising preventive early intervention for PTSD, specifically for individuals with high acute PTSD symptoms.

## 4. General discussion

### 4.1. Integration of findings

#### 4.1.1. Clinical and functional neuroimaging studies

There is a large body of literature that indicates that oxytocin administration beneficially affects neurobiological and socio-emotional vulnerability factors for PTSD that are likely etiologically involved in PTSD development (chapter 2; Frijling et al., 2014b). We hypothesized that intranasal oxytocin administration early posttrauma could reduce PTSD risk by attenuating amygdala hyperreactivity to fearful stimuli and by enhancing amygdala-centred functional connectivity of emotion regulation networks (i.e. amygdala-ventral PFC functional connectivity), especially in response to trauma-related stimuli. In line with our hypothesis, we observed that repeated oxytocin administration reduced PTSD symptoms up to six months posttrauma in recently trauma-exposed emergency department patients with high acute PTSD symptoms (chapter 7; van Zuiden et al., 2016). Contrary to our hypothesis, however, we demonstrated that a single intranasal oxytocin administration acutely increased neural fear processing (chapter 4; Frijling et al., 2016a) and potentially impeded neural emotion regulation (chapter 5; Frijling et al., 2016b) in recently trauma-exposed individuals. Based on our neuroimaging findings, we initially suggested that caution was warranted in administering intranasal oxytocin in recently trauma-exposed individuals (chapter 5; Frijling et al., 2016b). However, given the results of our clinical study – which were available at a later time point than the neuroimaging results – we eventually concluded that intranasal oxytocin administration is a promising novel preventive intervention in recently trauma-exposed individuals at increased PTSD risk due to high acute PTSD symptoms (chapter 7; van Zuiden et al., 2016).

There are several explanations for the discrepancy between the beneficial oxytocin effects in our clinical study and the seemingly adverse oxytocin effects in the neuroimaging study. First, considering the salience processing theory as explanation of context dependent oxytocin effects (chapter 3; Olff et al., 2013) (see section below for more detailed discussion), it may be argued that the context of the neuroimaging study (e.g. unfamiliarity with the scanner and procedures, small space, limited light, anticipation of the trauma script) was likely perceived as unsafe or threatening by our participants, who were already distressed by their recent traumatic experience. Consequently, oxytocin-induced increased salience processing for these threat-related signals during scanning may have resulted in acute anxiogenic effects in our neuroimaging studies, which may be inferred from our observations of increased amygdala reactivity to fearful faces, generally decreased amygdala-vmPFC functional connectivity, decreased amygdala-vlPFC functional connectivity in response to the trauma script, and increased flashback intensity during the trauma script after oxytocin administration (chapters 4, 5; Frijling et al., 2016a, 2016b). In contrast, administering oxytocin in the safe home environment may have enhanced positive perceptions of safety signals associated with the familiar home environment, leading to reduced anxiety – and PTSD symptoms – in the long-term (chapter 7; van Zuiden et al., 2016).

Second, the effects of oxytocin administration on either neural functioning or clinical outcome measures associated with anxiety may depend on oxytocin administration frequency (i.e. single versus repeated administration). An administration frequency dependent effect on anxiety is a well-known phenomenon for selective serotonin reuptake inhibitors (SSRIs). A single SSRI administration acutely increased fear learning in rodents and may potentiate anxiety in humans (for review see Burghardt & Bauer, 2013) – a process that that is dependent on SSRI effects on amygdala function (Ravinder, Burghardt, Brodsky, Bauer, & Chattarji, 2013); chronic SSRIs administration reduces anxiety and is therefore used to treat anxiety disorders (Baldwin et al., 2014). In high anxiety rats, chronic central oxytocin administration for six days reduced anxiety-related behaviour, whereas there was no effect on anxiety-related behaviour after a single administration (Slattery & Neumann, 2010). Furthermore, differential effects of a single, repeated (i.e. four administrations over seven days) and chronic subcutaneous oxytocin administrations on memory consolidation and fear-related behaviour were recently observed in a rat model of PTSD. In this model, rats were shocked and subsequently re-exposed to the shock context without the shock two, five, and seven days later, during which contextual fear behaviour was assessed. Generalized fear behaviour was assessed two weeks after shock exposure. Although a single administration of oxytocin immediately after shock exposure enhanced contextual fear behaviour two days later without affecting subsequent fear behaviour, repeated and chronic subcutaneous oxytocin administration for seven to 14 days initiated after shock exposure reduced generalized fear behaviour at 14 days after shock exposure (Janezic et al., 2016). As the increase in contextual fear behaviour after a single oxytocin administration immediately after shock exposure may represent an oxytocin-induced increase in (fear) memory consolidation, the authors suggest that the long-term anxiolytic effect of repeated and chronic oxytocin administration may be the result of an oxytocin-mediated increase of (extinction) memory consolidation during re-exposure to the trauma context in safe conditions (Janezic et al., 2016). These observations are in line with findings in rodents (Zoicas, Slattery, & Neumann, 2014) and humans (Acheson et al., 2013; Eckstein et al., 2015) indicating that oxytocin administration prior to fear extinction increases fear extinction (recall) (but see also Acheson, Feifel, Kamenski, Mckinney, & Risbrough, 2015; Eskandarian et al., 2013; Toth, Neumann, & Slattery, 2012; for contrasting results).

Third, it may be suggested that intranasal oxytocin administration does not reduce PTSD symptoms by affecting amygdala function and anxiety as primary mechanism; the beneficial effects of oxytocin administration on PTSD symptom development may be related to its effects on other vulnerability or etiological factors for PTSD. Repeated oxytocin administration may have increased social support seeking behaviour (Cardoso, Valkanas, Serravalle, & Ellenbogen, 2016; Preckel et al., 2014) – potentially by affecting neural reward functioning (Groppe et al., 2013; e.g. Nawijn et al., 2016a, 2016b) – or may have affected autonomic or glucocorticoid stress reactivity (Cohen et al., 2010). Considering the latter pathway, in rats it was previously observed that central oxytocin administration to the hippocampus immediately after exposure to a reminder of a severe stressor reduced PTSD-like behaviour one week later, which was accompanied by an oxytocin-induced decrease in glucocorticoid receptor (GR) expression in the hippocampus (Cohen et al., 2010). As PTSD vulnerability is associated with pre-existing high GR number and GR-sensitivity to glucocorticoids (van Zuiden et al., 2013), oxytocin administration may attenuate the negative effects of high GR-sensitivity associated with PTSD vulnerability by downregulating the total number of GRs expressed. Of note, glucocorticoid signalling in the hippocampal-medial PFC circuitry mediates memory contextualization (Liberzon & Abelson, 2016; Van Ast, Cornelisse, Meeter, Joëls, & Kindt, 2013). Memory contextualization refers to storing memories within the original encoding context, which may prevent memory generalization. Memory generalization is a process which is likely augmented in PTSD (Elzinga & Bremner, 2002) which can lead to trauma-related fear responses to neutral stimuli, potentially as a consequence of inadequate memory contextualization (Liberzon & Abelson, 2016). Deficits in hippocampal-mPFC circuit dependent contextual processing and associated aberrant glucocorticoid stress reactivity may thus be an important psychobiological correlate of PTSD (vulnerability), which may be potentially modified by oxytocin administration affecting hippocampal GR expression. The results of the study discussed above showing increased acute contextual fear but reduced more long-term generalized fear behaviour in severely stressed rats (Janezic et al., 2016) may also be explained in light of this hypothesis. Therefore, oxytocin administration effects on glucocorticoid stress reactivity and associated hippocampal-mPFC circuit dependent memory contextualization could be a neurobiological mechanism underlying the observed beneficial effects of oxytocin administration early after trauma on subsequent PTSD symptom severity.

Although we did observe long-term reduced anxiety in parallel to decreased PTSD symptoms in our clinical study, it remains unclear whether this effect is mediated by the initially hypothesized oxytocin effects on amygdala function. However, the neuroimaging results add important additional insights into acute effects of a single administration of oxytocin on amygdala function. We were not the first to report increased amygdala reactivity and increased amygdala-PFC functional connectivity after intranasal oxytocin administration. In healthy women oxytocin administration increased amygdala reactivity to emotional stimuli (Domes et al., 2010; Lischke et al., 2012). The healthy control groups in studies with patients with PTSD, generalized social anxiety disorder and borderline personality disorder also showed increased amygdala reactivity (Bertsch et al., 2013; Koch et al., 2016a), decreased amygdala-medial PFC functional connectivity (Dodhia et al., 2014), or no effect on amygdala function (Labuschagne et al., 2010) after oxytocin. As our sample consisted of recently trauma-exposed individuals without a current PTSD diagnosis, our results may be best comparable to results of the healthy trauma-exposed control group in the PTSD study, which showed increased amygdala reactivity to emotional faces after oxytocin (Koch et al., 2016a).

Taken together, the results of our clinical study provide stronger evidence than our neuroimaging study findings of our conclusion that intranasal oxytocin is a promising novel preventive intervention for PTSD. Our findings encourage further research into the clinical efficacy and feasibility of repeated oxytocin administration for PTSD prevention. However, it remains unclear whether observed long-term clinically beneficial effects are mediated by the initially hypothesized oxytocin effects on amygdala function. It may be argued that there is an administration frequency dependent effect of oxytocin on anxiety and amygdala function in recently trauma-exposed individuals, or that the beneficial effects or oxytocin are mediated by oxytocin effects on glucocorticoid-signalling dependent context processing.

#### 4.1.2. Explaining context and interindividual differences dependent effects of intranasal oxytocin administration

As hypothesized (chapters 2, 3, 4; Frijling et al., 2016a, 2016b; Olff et al., 2013), we observed context and/or interindividual differences dependent effects of oxytocin administration in all our intranasal oxytocin studies. In our clinical study, oxytocin reduced PTSD symptom development only in individuals with high acute PTSD symptoms (chapter 7; van Zuiden et al., 2016). In our emotional face-matching fMRI paradigm, we found both stimulus valence (i.e. context) and sex (i.e. interindividual) dependent effects of oxytocin on amygdala reactivity (chapter 4; Frijling et al., 2016a). We also observed an oxytocin-induced attenuation of amygdala left-vlPFC functional connectivity after a trauma reminder only, not after the neutral script (i.e. context effect depending on script-condition valence) (chapter 5; Frijling et al., 2016b). These differential effects of oxytocin administration may be explained by oxytocin enhancing salience processing (chapter 3; Olff et al., 2013). The salience theory of oxytocin administration effects postulates that in contexts perceived as positive, supportive or safe, oxytocin will enhance the salience safety signals, consequently attenuating stress and anxiety. In unpredictable threatening situations however, oxytocin will increase salience of threat signals, and increase stress and anxiety (chapter 3, Olff et al., 2013; Shamay-Tsoory & Abu-Akel, 2016). Interindividual differences (e.g. attachment representations, childhood trauma, prior psychopathology, sex) may affect the initial perception of a given a context as safe or threatening.

In our study describing results of the face-matching paradigm (chapter 4; Frijling et al., 2016a), we argued that it is likely that individuals who were recently exposed to a potentially life threatening event experience fear-related stimuli as more salient than neutral and happy stimuli; a response that may be further enhanced as result of oxytocin-induced increased salience processing. However, in our amygdala-centred functional connectivity paradigm (chapter 5; Frijling et al., 2016b), we concluded that the observed effects of oxytocin administration did not fit the salience processing theory, as oxytocin increased amygdala-posterior insula functional connectivity, whereas anterior insula function is generally implicated in salience processing (Menon & Uddin, 2010; Seeley et al., 2007). Of note, we did not investigate whether oxytocin administration affected (anterior) insula functional reactivity, which would have provided important additional information with respect to oxytocin administration effects on salience processing. Therefore, rejecting the salience theory solely based on the lack of an oxytocin-induced increase in amygdala-anterior insula functional connectivity may be premature. It may be argued that the unfamiliar scanner context, and (the anticipation of) the trauma-script, may have resulted in perceiving the context as threatening. This could have led to oxytocin-induced enhanced salience of threat-related stimuli and thus seemingly anxiogenic neural oxytocin effects in our amygdala-centred functional connectivity paradigm (chapter 3; Olff et al., 2013). The interindividual differences dependent effect of oxytocin in our clinical study, i.e. a beneficial oxytocin effect on PTSD symptom development in individuals with high acute PTSD symptom severity only, is in line with previous observations that intranasal oxytocin effects depend on the presence and/or severity of psychiatric symptom (Bertsch et al., 2013; Cardoso et al., 2014; Dodhia et al., 2014; Koch et al., 2016a; Nawijn et al., 2016b).

It should be noted that the salience theory was based on a large body of literature on the effects of a single intranasal oxytocin administration on (predominantly) socio-emotional functioning; there is no direct evidence that the salience theory also holds for repeated oxytocin administration effects. An alternative suggestion for previously observed differential effects of oxytocin administration between healthy individuals and patients with a psychiatric disorder is that oxytocin administration may only have beneficial effects in individuals who have something to gain with regard to social or emotional functioning (Macdonald & Feifel, 2013; Weisman & Feldman, 2013) and associated neurobiological processes.

Taken together, oxytocin administration effects that depend on context and interindividual differences as observed in our studies still fit with the model that oxytocin increases salience processing, but may also result from other processes that have yet to be further elucidated. To further evaluate the validity of the salience processing model, for explaining (repeated) oxytocin administration effects on reducing PTSD symptom development, (perception of) context factors should be explicitly assessed and tested as moderators of oxytocin effects in future studies.

### 4.2. Limitations

Although we were the first to study intranasal oxytocin effects in recently trauma-exposed individuals, meanwhile conducting the largest clinical RCT with intranasal oxytocin to date, some limitations of our studies need to be addressed. First, the statistical power of our clinical study was limited. We halted our study halfway, as our pre-planned interim analysis indicated low conditional power to detect a significant overall effect of oxytocin administration on PTSD symptoms at our primary outcome (1.5 month posttrauma follow-up). As results from studies with low statistical power are at increased risk for Type I error, replication of our findings is clearly warranted. In addition, the sample size of our imaging studies was also modest, therefore it was not possible to reliably test whether the effects of oxytocin administration on amygdala function were moderated by interindividual differences as we observed in our clinical study. We did explore whether acute PTSD symptom severity was differently associated with amygdala reactivity to fearful faces and found no differential effect (chapter 4; Frijling et al., 2016a), but it should be noted that the sample size was small for such analysis. Because we were limited in statistical power, we could not investigate sex-differential effects of oxytocin, even though there are indications that oxytocin administration has differential (neural) effects in men and women (Koch et al., 2016b; Rilling et al., 2014). Also, as over 80% of our participants experienced an accidental trauma, we were not able to investigate whether type of trauma exposure was associated with differential oxytocin effects. Previously, rape victims benefited more from exposure therapy initiated immediately after trauma than individuals exposed to other trauma types (Rothbaum et al., 2012) and oxytocin administration in rodents promoted fear extinction of social fear (Toth et al., 2012), but impaired fear extinction of non-social fear (Zoicas et al., 2014). Further, as we did not have sufficient participants taking part in both the fMRI and clinical study, we were not able to investigate whether acute effects of a single oxytocin administration on amygdala function were in fact related to PTSD symptom development (after repeated administration). An additional limitation of the neuroimaging study is that we did not scan a recently trauma-exposed group with low levels of initial distress, which would have aided in interpreting our neuroimaging results, i.e. whether oxytocin effects were likely beneficial or not.

### 4.3. Future perspectives

First, as we were the first to assess intranasal oxytocin effects in recently trauma-exposed individuals, our results should be replicated in similar and different samples of recently trauma-exposed individuals. Second, it should be investigated whether effects of repeated oxytocin administration on PTSD symptom development are mediated by oxytocin effects on autonomic and glucocorticoid stress reactivity (and associated contextual processing), functional fear neurocircuitry and/or other socio-emotional processes (e.g. related to reward functioning).

Furthermore, considering the potential future use of intranasal oxytocin in routine clinical practice – for PTSD prevention but for also other psychiatric indications – it is highly desirable to better understand when beneficial, null or even potentially non-beneficial effects of oxytocin administration can be expected. Particularly, oxytocin effects on fear memory consolidation and fear extinction should be studied, as there are indications that oxytocin-mediated enhanced fear and extinction memory consolidation may have either anxiolytic or anxiogenic effects, depending on administration frequency and timing in relation to fear conditioning (Janezic et al., 2016; Toth et al., 2012), and whether the conditioned stimulus is social or not (Toth et al., 2012; Zoicas et al., 2014). How intranasal oxytocin affects fear consolidation and fear extinction may have implications for determining the optimal timing, and duration of intranasal administration in clinical settings. Additionally, considering the salience processing theory, (the perception of) contextual factors moderating oxytocin administration effects on short and long-term anxiety-related outcome measures should be better understood. Furthermore, dose-response effects of oxytocin administration on anxiety should be further investigated: in mice it was demonstrated that a high dose of (chronic) oxytocin administration increased anxiety, whereas a low dose (chronic) oxytocin administration had the opposite effect (Peters, Slattery, Uschold-Schmidt, Reber, & Neumann, 2014). This dose-dependent effect may be mediated by oxytocin binding to arginine vasopressin receptors, which has been associated with enhanced fear expression and anxiety (Huber et al., 2005), but the exact mechanisms explaining these dose-dependent effects of oxytocin on anxiety are not yet understood.

In addition, a better understanding of the pharmacodynamics of intranasally administered oxytocin is needed. Oxytocin has a half-life of 3–20 min in blood, and the peptide is immediately degraded upon oral ingestion. There is no clear consensus on if and how intranasally administered oxytocin reaches the brain. Although intranasal oxytocin administration increased salivary (van IJzendoorn, Bhandari, van der Veen, Grewen, & Bakermans-Kranenburg, 2012), plasma (Striepens et al., 2013) and cerebrospinal fluid (Striepens et al., 2013) concentrations of oxytocin, it remains unknown whether these observations reflect amplified endogenous oxytocin release upon intranasal oxytocin administration (potentially as a result of positive feedback mechanisms (Ludwig, 2014; van IJzendoorn et al., 2012)) or the exogenously administered compound. However, recent studies in which oxytocin was administered both intravenously and intranasally suggest a direct nose-to-brain route for oxytocin (Quintana, Alvares, Hickie, & Guastella, 2015; Quintana et al., 2016). In addition, the exact duration of oxytocin administration effects on brain function, stress reactivity and socio-emotional behaviour remain unknown, although it was recently demonstrated that neural effects of intranasal oxytocin may be observed up to at least 80 min post-administration (Paloyelis et al., 2014).

If our findings are replicated, intranasal oxytocin may be a safe and cheap preventive intervention for PTSD (MacDonald et al., 2011). However, another important avenue for future research is whether intranasal oxytocin can be implemented in routine clinical practice as preventive intervention for PTSD. Within our research setting we have shown that it is feasible to screen a large number of – but not all – trauma-exposed emergency department patients early after trauma, to initiate the intervention and to provide follow-up assessments. However, it is unclear whether these actions and responsibilities can be translated to clinical practice and whether this would be cost-effective. Additionally, we used the trauma screening questionnaire (TSQ) (Brewin et al., 2002) and peritraumatic distress inventory (PDI) (Brunet et al., 2001) early posttrauma to identify and include trauma-exposed individuals at increased PTSD risk within one to seven days posttrauma. As these measures of early posttrauma distress did not moderate the effects of oxytocin on PTSD symptom development (potentially due to its limited specificity for PTSD when applied within the first week posttrauma), they are not suitable to determine which trauma-exposed individuals should be offered an intranasal oxytocin intervention early posttrauma. As administering the clinician-administered PTSD scale (CAPS) to (a selection of) trauma-exposed individuals is likely not feasible in clinical practice – considering the duration of the interview – other clinically applicable screeners for increased PTSD risk and moderators of intranasal oxytocin effects on PTSD symptom development should be assessed before intranasal oxytocin administration for the prevention of PTSD may eventually be feasible in clinical practice. Novel technology solutions to screen for increased risk for PTSD that can be more easily implemented at emergency departments (and other trauma settings) could be of help in future (implementation) studies on early preventive interventions for PTSD (Olff, Van Zuiden, & Bakker, 2015).

Finally, there is only limited evidence linking functioning of the oxytocin system directly to PTSD risk (Lucas-Thompson & Holman, 2013). In addition, the neurocircuitry model of PTSD vulnerability as proposed by Admon, Milad, and Hendler (2013) is largely based on indirect evidence from studies demonstrating associations between genetics and/or childhood trauma – both risk factors for (adulthood) PTSD – and neural abnormalities. Therefore, prospective studies should assess whether pre- or early posttrauma measures of oxytocin system functioning and the structure and function of the fear neurocircuitry predict subsequent PTSD development, as knowledge on neurobiological risk factors that may be etiologically involved in PTSD development may yield new targets for preventive interventions for PTSD.

## 5. Conclusion

We observed that repeated intranasal oxytocin administration early posttrauma reduced subsequent PTSD symptom development in recently trauma-exposed emergency department patients with high acute PTSD symptoms. Although replication is necessary, these findings indicate that intranasal oxytocin is a promising novel preventive intervention for individuals at increased risk for PTSD due to high acute symptom severity. However, we also observed acutely increased neural fear processing and impeded neural emotion regulation after a single oxytocin administration in recently trauma-exposed individuals. It remains unknown whether the positive clinical effects after repeated oxytocin administration are mediated by oxytocin effects on amygdala function, or whether the beneficial effects in our clinical study are mediated by other neurobiological and/or socio-emotional processes. Administration frequency dependent effects of oxytocin may be related to these seemingly contrasting effects on anxiety-related measures in our clinical and neuroimaging studies. In addition, the salience processing theory of oxytocin effects may serve as an explanatory framework for these seemingly contrasting results between the two study paradigms. Clinically relevant contextual and interindividual moderators of oxytocin effects should be investigated in the future, as well as underlying neurobiological and socio-emotional mechanisms that mediate oxytocin effects on anxiety-related outcomes and the development of PTSD symptoms. In all, our findings encourage further research into the clinical efficacy and feasibility of repeated oxytocin administration as early preventive intervention for PTSD, in order to try and reduce the high individual and societal burden associated with trauma exposure and PTSD.

## Highlights

- Oxytocin administration may prevent posttraumatic stress disorder (PTSD) by affecting PTSD vulnerability factors (e.g. neural fear processing).
- We investigated intranasal oxytocin effects on acute neural fear processing and subsequent PTSD symptoms in recently trauma-exposed individuals.
- A single oxytocin administration increased neural fear processing.
- Repeated oxytocin administration reduced PTSD symptoms up to six months posttrauma in participants with high acute PTSD symptoms, and is therefore a promising early preventive intervention for PTSD.
